# Effect of Ultrasonic Vibration on Structural and Physical Properties of Resin-Based Dental Composites

**DOI:** 10.3390/polym13132054

**Published:** 2021-06-23

**Authors:** Abdul Samad Khan

**Affiliations:** Department of Restorative Dental Sciences, College of Dentistry, Imam Abdulrahman Bin Faisal University, Dammam 31441, Saudi Arabia; akhan@iau.edu.sa; Tel.: +966-594781075

**Keywords:** dentistry, dental polymers, preheating, ultrasonic scaler, dental composites, degree of conversion, FTIR, principal component analysis, micro-CT, voids

## Abstract

This study aimed to investigate the influence of ultrasonic heat before photo-polymerization on the structural and physical properties of dental composites. Commercially available bulk-fill, nano-hybrid, micro-hybrid, and flowable composites were used. The samples were divided into three groups i.e., (i) without ultrasonic activation, (ii) ultrasonic activation at 15 Hz for 30 s, and (iii) ultrasonic activation at 15 Hz for 60 s. The degree of conversion percentage (DC%) and structural changes were evaluated with Fourier transform infrared spectroscopy. The presence of voids in restored tooth cavities were investigated with micro-computed tomography. The statistical analysis was performed using a one-way analysis of variance (ANOVA) post hoc Tukey’s test. The DC% was significantly increased with ultrasonic application in all groups except for flowable composites, whereby flowable composite showed a significant increase with 30 s ultrasonic activation only. The highest DC% was observed in 60 s ultrasonically activated nano-hybrid and micro-hybrid composites. The voids were reduced linearly with ultrasonic application in flowable and bulk-fill composites; however, non-linear behavior was observed with micro-hybrid and nano-hybrid composites, whereby the difference was significant within the groups. The frequency and time of the ultrasonic application is an important factor to consider and can be used to preheat composites before clinical application.

## 1. Introduction

Many patients are looking for dental restorations other than amalgam restorations mainly because of esthetic reasons [1]. In October 2013, a new international binding treaty instrument called the Minamata Convention on Mercury opened for signature [2]. There is a strong perception that eventually mercury-based amalgam restorations will phase out from clinical practices. Therefore, resin-based composites are most preferred among patients and clinicians [3]. Almost five hundred million resin-based restorations are used per year [4]. Modern resin composite has evolved and enables clinicians to provide restorations that are both esthetic and biologically conservative [5]. However, the major limitations associated with resin-based composites are polymerization shrinkage, microleakage, and the presence of unreactant monomers [6,7].

Various types of resin-based composite materials are available these days for direct restorative methods. The most common types are micro- and nano-hybrid composites, however, hybrid composites with a high concentration of fillers may cause stress and may not adequately adapt to an internal area of the cavity and leave behind gaps [8]. The incremental technique was introduced to overcome these issues; however, this technique is sensitive and time consuming [9]. Flowable composites were initially introduced in the late 1990s as lining material [10] and are intended for use as a base below a layer of hybrid composite [11]. Bulk-fill composites are considered to be used in larger increments i.e., up to 4 mm [12]. The low viscosity of bulk-fill composites was achieved by increasing resin monomer content from 20 to 40 wt.% and decreasing the filler loading i.e., almost 60–68 wt.% [13]. Despite the ease of use, polymerization shrinkage and low mechanical properties are inevitable drawbacks of flowable and bulk-fill composites [14,15]. Therefore, clinicians are still skeptical to use these materials as direct restorative material under the masticatory load. Recent advancements in bulk-fill flowable composites allow them to be placed directly without a traditional nano/micro-hybrid composite capping layer [16].

It has been observed that after photo-activation of resin-based composites the polymerization reaction is not complete and unreactant monomers are released and may cause local adverse effects [17,18]. The presence of unpolymerized monomers is linked with the degree of conversion (DC), whereby DC of various composites ranges between 35 and 77% [19,20]. Low DC might be due to the restricted mobility of radical chain ends and pendant methacrylate groups [21]. Consequently, they affect the mechanical and physical properties such as wear resistance, hardness, and water sorption [22].

With regards to resin-based restoration improvement, studies have focused on preheating [23,24]. Preheating of composites enhances DC and marginal adaptation [25,26], which could be due to the lower viscosity of the material because of a heating process. The process can increase the degree of movements of the molecules leading to better cross-linking and higher influence [27,28]. One of the sources of thermal stimulation is sonic and ultrasonic scalers. The ultrasonic vibration is created by two methods i.e., magnetic and piezoelectric methods [29]. The piezoelectric method transfers higher efficiency of energy and vibration and consequently reduces energy consumption and the rise of temperature during the transferring process [30].

Ultrasonic scalers have been used in a variety of dental applications. The known functions of ultrasonic scalers are for preventive, operative, endodontic, prosthetic, and orthodontic procedures [31,32,33]. It is reported that using an ultrasonic tip might improve the properties of glass ionomer cement properties due to the generation of heat [34,35]. Studies showed that the ultrasonic application increased the intrapulpal temperature; however, the increase remains below the critical value of 5.5 °C [36].

The authors could not find any study where the ultrasonic application was applied on resin-based composites to evaluate the DC and changes in porosities. Therefore, this study is aiming to achieve a restorative composite with improved polymerization kinetic reaction via the influence of ultrasonic heat and vibration before photo-polymerization of these materials. It is hypothesized that ultrasonic heat and vibration would increase the mobility of monomer molecules within a matrix which in turn enhances the degree of conversion and reduce the void volumes of various resin-based composites.

## 2. Materials and Methods

In this experimental study, four commercially available light-cured resin-based materials were investigated. These resin-based materials belonged to the bulk-fill composite, nano-hybrid composite, micro-hybrid composite, and flowable composite categories. The detailed composition of these materials is given in Table 1.

Samples were divided into three groups based on preparation methodology i.e., Group 1, control group (CT): specimens (commercial composites) were cured using LED light cure (wavelength ~ 480 nm; intensity ~ 1250 to 2000 mW/cm^2^; Mini LED, OEM, Satelec, Viry-Châtillon, France) without application of ultrasonic scaler; Group 2 (US-30): specimens were first activated with ultrasonic scaler tip (PiezoLED Scaler Tips-202, Piezo ultra scaler, PiezoLED, Kavo, Warthausen, Germany) without water for 30 s at 15 Hz, then cured using LED light cure; Group 3 (US-60): specimens were first activated with ultrasonic scaler tip without water for 60 s at 15 Hz, then cured using LED light cure.

### 2.1. Sample Preparation

After being granted ethical permission from the institutional research unit committee, sixty caries- and fracture-free extracted human teeth (molars) were collected and were disinfected with 70/30 ethanol for 10 min [40]. The disinfected teeth were stored in normal saline at 4 °C (not for more than a month) [41]. A conventional class V cavity (4 mm mesio-distal width, 2 mm occluso-gingival height and 2 mm deep) [42] was prepared using diamond fissure burs (836, 856, midwest^®^, Dentsply Sirona, Charlotte, NC, USA) on the buccal and lingual side of each tooth by a single operator. A new bur was used after every five cavity preparations. The sample size was calculated as per World Health Organization’s specifications version 2.0.21 [43] keeping the power of study equal to 90% and the level of significance equal to 5%.

For a change in temperature analysis, the cavities were restored with composites of each group (*n* = 6), whereby, etching and bonding processes were not performed. In Group 1 a K-type thermocouple (TES-1310 digital thermometer TES electrical electronic corp., Taipei, Taiwan) was used to record the temperature before curing. The tip of the thermocouple sensor was placed to record the temperature at two different areas within the restored cavity. For Group 2, before curing, composites were activated with an ultrasonic scaler tip for 30 s. The scaler tip was placed on the surface of composites. The temperature was recorded for each composite group, then composites were cured with LED for 40 s [42]. The same procedure was applied for Group 3 where composites were activated with 60 s ultrasonic application and temperatures were recorded for each composite group.

### 2.2. Degree Of Conversion Analysis

For a degree of conversion (DC) analysis, the prepared tooth cavity was used as mold and cavities were restored and composites were treated with an ultrasonic scaler (30 s and 60 s) and without ultrasonic application. Samples from each group were taken and placed on a diamond window and Fourier transform infrared spectroscopy (Nicolet iS50, Thermo Scientific, Waltham, MA, USA) was used to evaluate the degree of conversion. The resolution was set at 8 cm^−1^ with 64 scan numbers. The data were obtained at the 4000–525 cm^−1^ range. The spectrum of each sample (*n* = 10) was taken in uncured form, then samples were cured with LED for 40 s, and spectra were taken again. The tip of the light-curing device was as close as 1.0 mm to the surface of a material [44]. The degree of conversion was evaluated by using the formula;

DC% = 100 × [1 − (R_Polymerized_/R_Unpolymerized_)] (1)

where R is the ratio of the peak height of polymerized aliphatic to polymerized aromatic and unpolymerized aliphatic to unpolymerized aromatic groups. The aliphatic and aromatic peaks were recorded at 1638 cm^−1^ and 1608 cm^−1^, respectively. Due to the lack of aromatic C=C group in the SDR resin composite, the internal standard peak at 1602 cm^−1^ was used.

### 2.3. Methodology For Spectral Data Processing

Chemometric methods were used to quantify the spectral differences of various groups present in the data. These methods were performed using Unscrambler X 10.2 software, purchased from Camo software (Oslo, Norway). Pre-processing was comprised of baseline correction and unit vector normalization. Principal component analysis (PCA) was used to visualize systemic variation in data comprising of various cured and uncured dental composites spectra. The method reduces data dimensions by projecting it down to a lower-dimensional space in directions showing the largest variance. Variance in each direction is described by a principal component (PC) and a scores plot shows the distribution of spectra in relation to the PC. The PCA was performed over the complete spectral range, 600–3400 cm^−1^, 900–1200 cm^−1^, 1590–1650 cm^−1^, and 1700–1750 cm^−1^. Cluster analysis (CA) was performed over a full spectral range (600–3400 cm^−1^) by Ward’s method using squared Euclidean distance [45].

### 2.4. Micro-Computed Tomography (µ-CT) Analysis

The collected teeth were randomly divided into three major groups (*n* = 60), then each group was further subdivided into four groups as per commercial composites. Standard class V cavity was prepared on the buccal and lingual sides of each tooth. The teeth with composite restorations of each group (*n* = 10) were scanned with a micro-CT Skyscan 1172 machine, Bruker, Kontich, Belgium with the parameters of source voltage (kV) = 100, source current (µA) = 100, image pixel size (µm) = 10, filter = Al + Cu, image format = TIFF, exposure (ms) = 2400, rotation step (deg) = 0.500, frame averaging = 3, random movement = 10, and 360 rotation was used. Then raw images (tiff format) of each sample were processed for reconstruction to create cross-sections with NRecon version: 1.6.4.8, Skyscan 2011 software that converts file type to bitmap. The CTAn version 1.11.10.0+ (64-bit), 2003-11 Skyscan software was used for the calculation of the voids’ volume in cubic millimeters (mm^3^) inside the composite filling. Using CTvol version 2.2.1.0 program, the samples were viewed for realistic 3D visualization and creation of 3D images.

### 2.5. Statistical Analysis

Statistical analysis was performed using the Statistical Package for the Social Sciences (SPSS) version 22 (IBM Software, Armonk, NY, USA). The Shapiro–Wilk test was done to test the normal distribution of data. Analysis of variance (ANOVA) was used to compare the degree of conversion (DC%) and void volumes among the groups, followed by an independent *t-test* and post hoc Tukey’s test. The level of significance was set at 0.05.

## 3. Results

**Figure 1:**
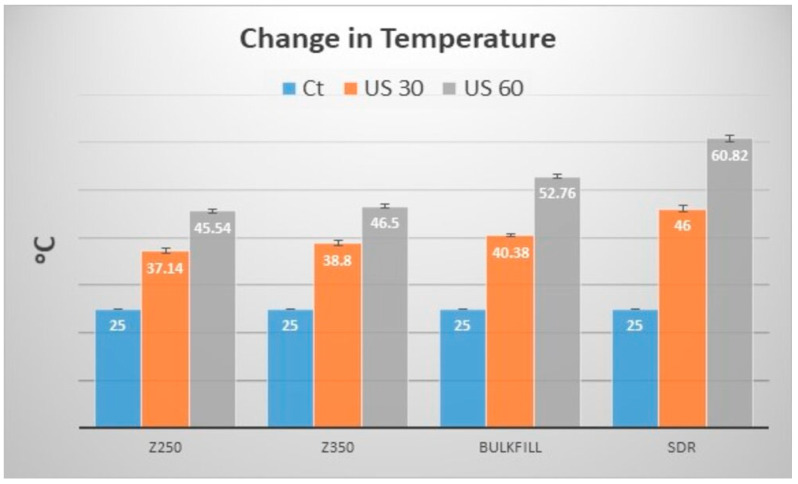
Change in temperature of composites i.e., Filtek Z250, Filtek Z350, Filtek Bulk-fill, and SDR with (30 s (US 30) and 60 s (US 60)) and without ultrasonic application (Ct).

The temperature change was recorded with ultrasonic application and the values are given in Figure 1. A significant difference (*p* = 0.000) was observed within and between groups after 30 s and 60 s of ultrasonic application. The SDR showed a maximum rise in temperature compared to other composites, where after 30 s and 60 s of ultrasonic application, the recorded temperature was 46 ± 0.62 °C and 60.82 ± 0.69 °C, respectively. The minimum rise in temperature was observed for Z250, whereby the recorded temperatures were 37.14 ± 0.57 °C and 45.54 ± 0.44 °C after 30 s and 60 s ultrasonic applications, respectively.

**Figure 2:**
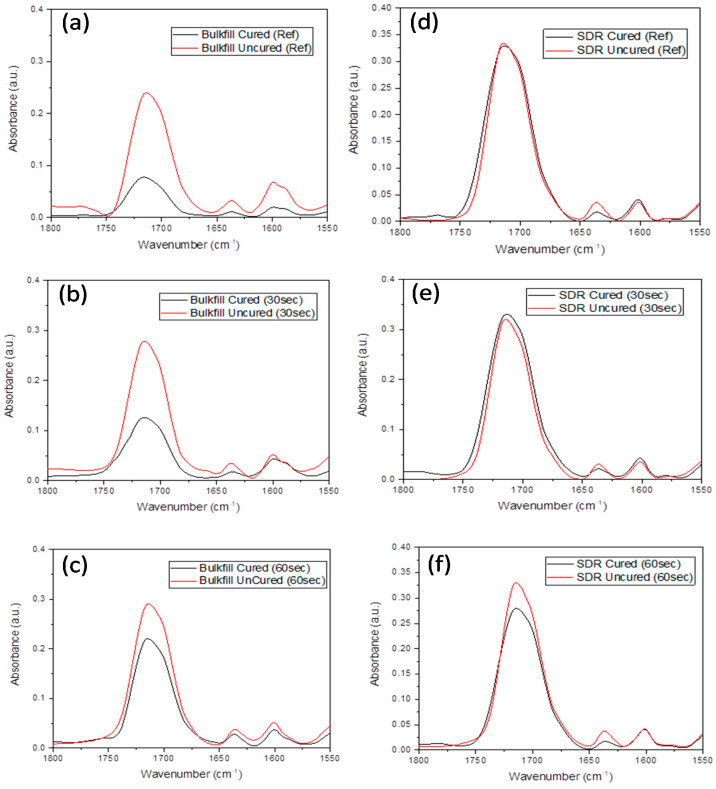
Comparative FTIR spectra of uncured and cured Bulk-fill composite (**a**) without ultrasonic application, (**b**) 30 s ultrasonic application, and (**c**) 60 s ultrasonic application. The comparative FTIR spectra of cured and cured SDR composite (**d**) without ultrasonic application, (**e**) 30 s ultrasonic application, and (**f**) 60 s ultrasonic application. The change in intensity was observed at 1715 cm^−1^ (C=O), 1635 cm^−1^ (aliphatic), and 1602 cm^−1^ (aromatic).

**Figure 3:**
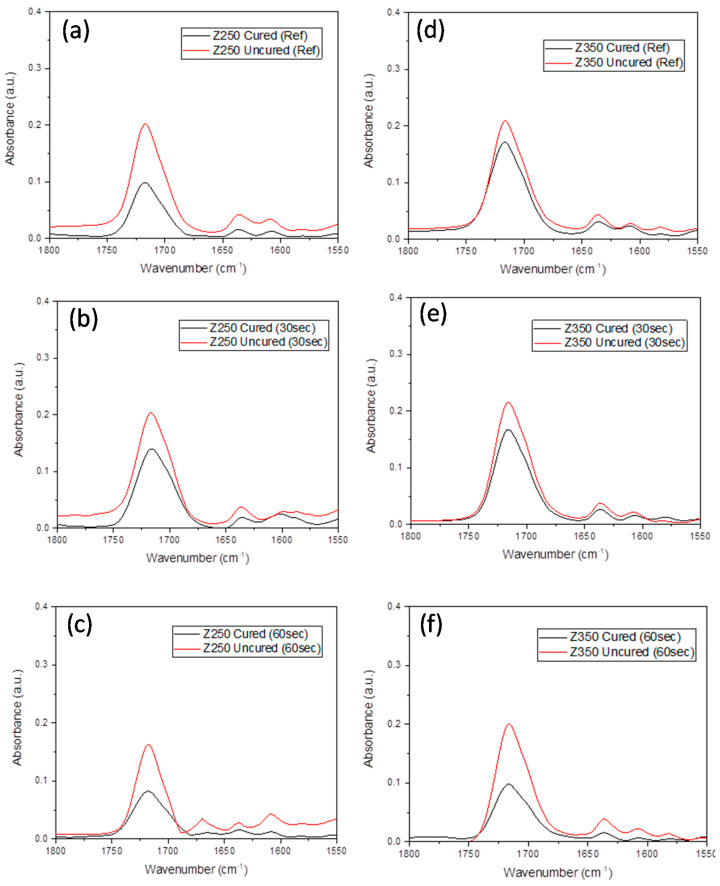
Comparative FTIR spectra of uncured and cured Filtek Z250 composite (**a**) without ultrasonic application, (**b**) 30 s ultrasonic application, and (**c**) 60 s ultrasonic application. The comparative FTIR spectra of cured and cured Filtek Z350 composite (**d**) without ultrasonic application, (**e**) 30 s ultrasonic application, and (**f**) 60 s ultrasonic application. The change in intensity was observed at 1715 cm^−1^ (C=O), 1635 cm^−1^ (aliphatic), and 1602 cm^−1^ (aromatic).

**Figure 4:**
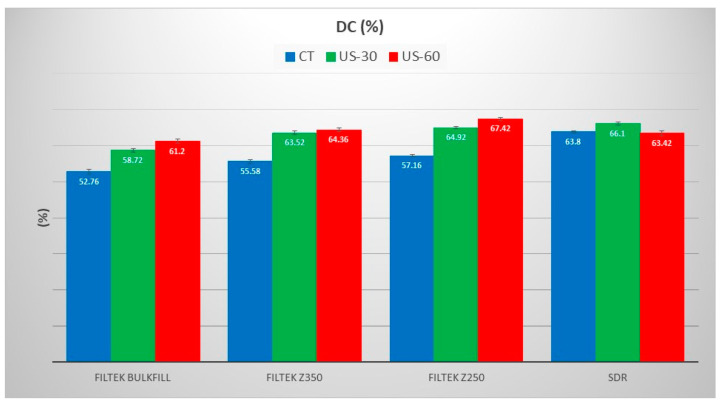
Mean (SD) DC% of composites (Filtek Bulk-fill, Filtek Z350, Filtek Z250, and SDR) without (CT) and with ultrasonic application for 30 s (US-30) and 60 s (US-60).

A significant difference was also observed between the 30 s and 60 s groups for both composites. The change in aliphatic and aromatic peaks of Z250 and Z350 composites with and without ultrasonic application is presented in Figure 2 and change in peak intensities at 1715 cm^−1^ (C=O), 1635 cm^−1^ (aliphatic), and 1602 cm^−1^ (aromatic) of Bulk-fill and SDR are given in Figure 3. Due to the lack of aromatic C=C group in the SDR resin composite, the internal standard peak at 1602 cm^−1^ was used. The peak intensities were reduced after curing. The DC% of all composite groups with and without application of the ultrasonic scaler is given in Figure 4. It was found that DC% was increased with an ultrasonic application for Bulk-fill, Z350, and Z250 composites, whereby a significant difference (*p* = 0.000) between control, 30 s, and 60 s groups was observed. SDR showed an increase in DC% with 30 s ultrasonic application and a significant difference between control and 30 s groups (*p* = 0.000). However, DC% was reduced with 60 s ultrasonic application and a non-significant difference was observed between control and 60 s groups (*p* = 0.336).

**Figure 5:**
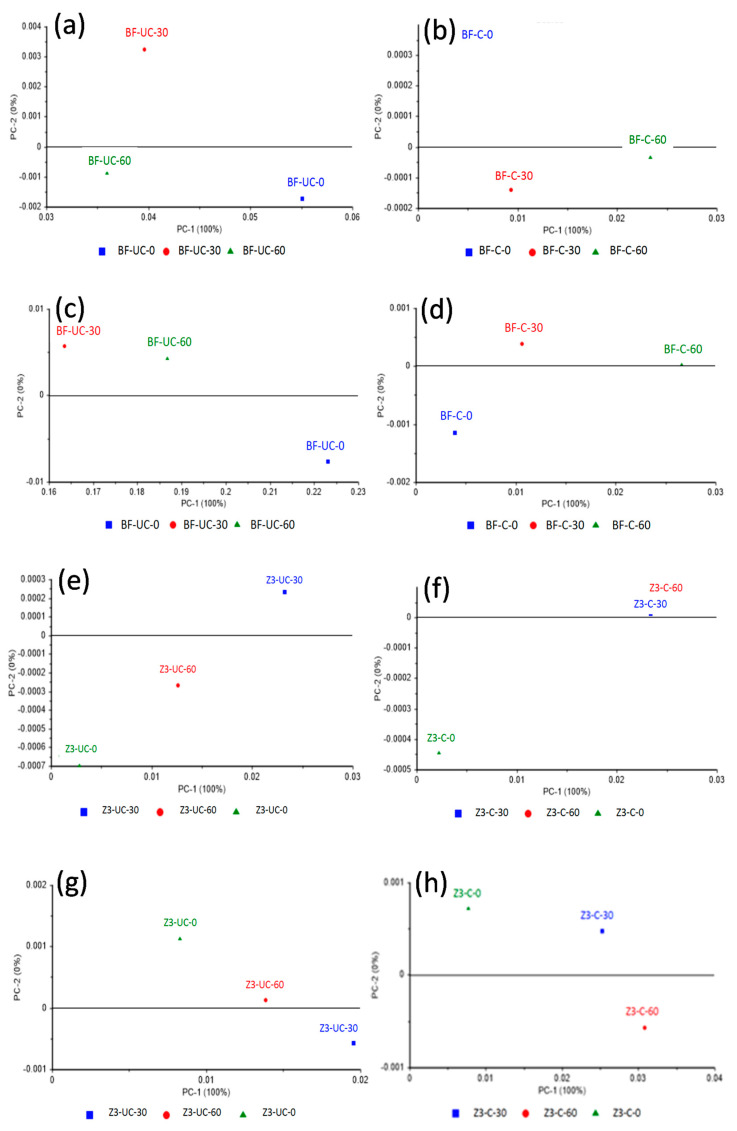
Principal component analysis of bulk-fill dental composite. (**a**) Uncured and (**b**) cured at spectral range 1590–1650 cm^−1^, (**c**) uncured and (**d**) cured at spectral range 1700–1750 cm^−1^. Principal component analysis of Filtek Z350 dental composite (**e**) uncured and (**f**) cured at spectral range 1590–1650 cm^−1^, (**g**) uncured and (**h**) cured at spectral range 1700–1750 cm^−1^. Both composites showed separation between control, 30 s, and 60 s of both cured and uncured phases at both the spectral ranges with 100% variance.

For Bulk-fill and Z350 composites, the complete spectral range and the 900–1200 cm^−1^ regions showed slightly less variance where the minimum variance of PC1 was 91% in a complete spectral range of uncured bulk-fill dental composites (data not shown). Bulk-fill and Z350 composites demonstrated (Figure 5) excellent separation between control, 30 s, and 60 s of both cured and uncured dental composites at both the spectral ranges (1590–1650 cm^−1^ and 1700–1750 cm^−1^) with 100% variance observed in the first component.

**Figure 6:**
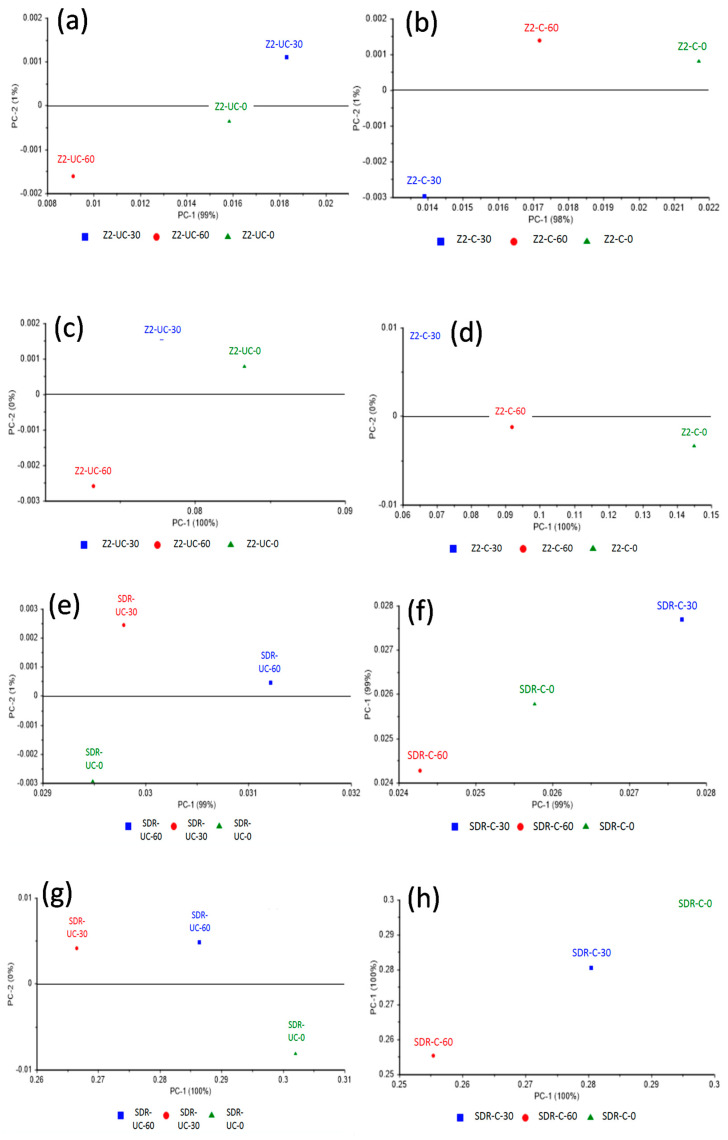
Principal component analysis of Filtek Z250 dental composite; (**a**) uncured and (**b**) cured at spectral range 1590–1650 cm^−1^, (**c**) uncured and (**d**) cured at spectral range 1700–1750 cm^−11^. Principal component analysis of SDR dental composite (**e**) uncured and (**f**) cured at spectral range 1590–1650 cm^−1^, (**g**) uncured and (**h**) cured at spectral range 1700–1750 cm^−1^. Both composites showed separation between control, 30 s, and 60 s of both cured and uncured phases at both the spectral ranges with 100% variance at 1700–1750 cm^−1^ and 98% and 99% variances at 1590–1650 cm^−1^ spectral range of cured and uncured SDR and Filtek Z250 dental composites, respectively.

SDR and Z250 also demonstrated (Figure 6) excellent separation between control, 30 s, and 60 s groups of both cured and uncured dental composites at both the spectral ranges (1590–1650 cm^−1^ and 1700–1750 cm^−1^). The spectral range at 1700–1750 cm^−1^ showed 100% variance in the first components of cured and uncured SDR and Z250 dental composites. The 1590–1650 cm^−1^ spectral range showed a minimum variance of 98% and a maximum of 99% in the first components of cured and uncured SDR and Z250 dental composites.

**Figure 7:**
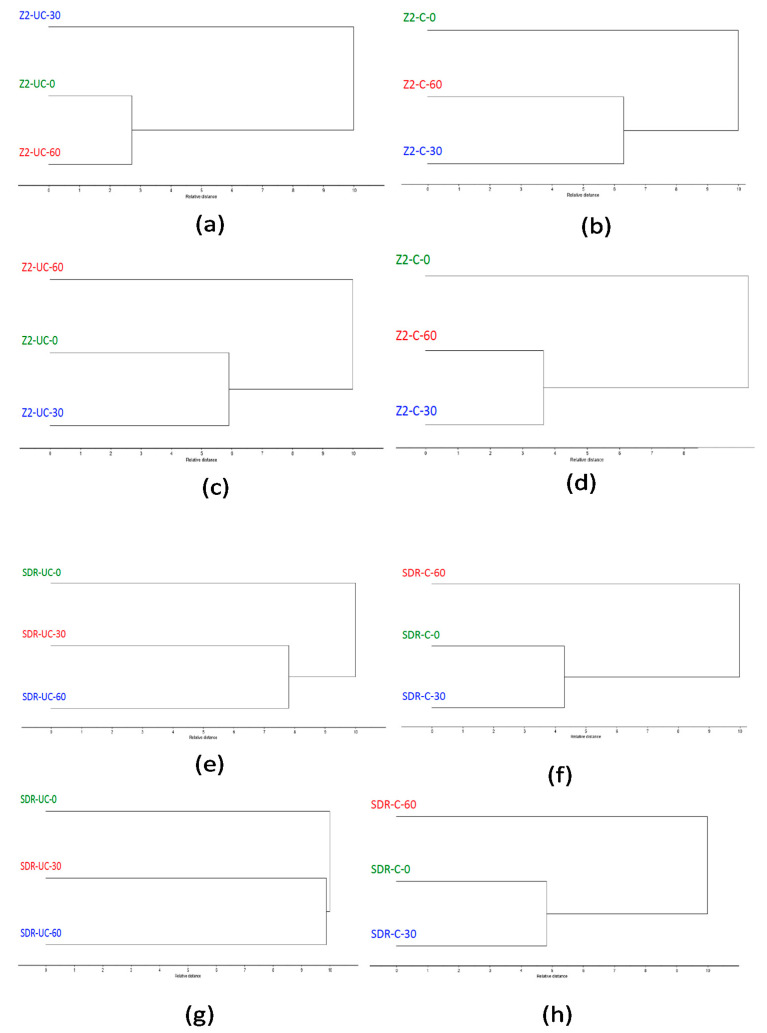
Cluster analysis of Filtek Z250 composite showing uncured and cured at spectral range 1590–1650 cm^−1^ (**a**,**b**), uncured and cured at spectral range 1700–1750 cm^−1^ (**c**,**d**). Cluster analysis of SDR composite showing uncured and cured at spectral range 1590–1650 cm^−1^ (**e**,**f**), uncured and cured at spectral range 1700–1750 cm^−1^ (**g**,**h**). At both the spectral ranges, there was a distinct separation without ultrasonic treatment from 30 s and 60 s while in the uncured state. However, once cured, control was branched closer to the 30 s treated composite.

**Figure 8:**
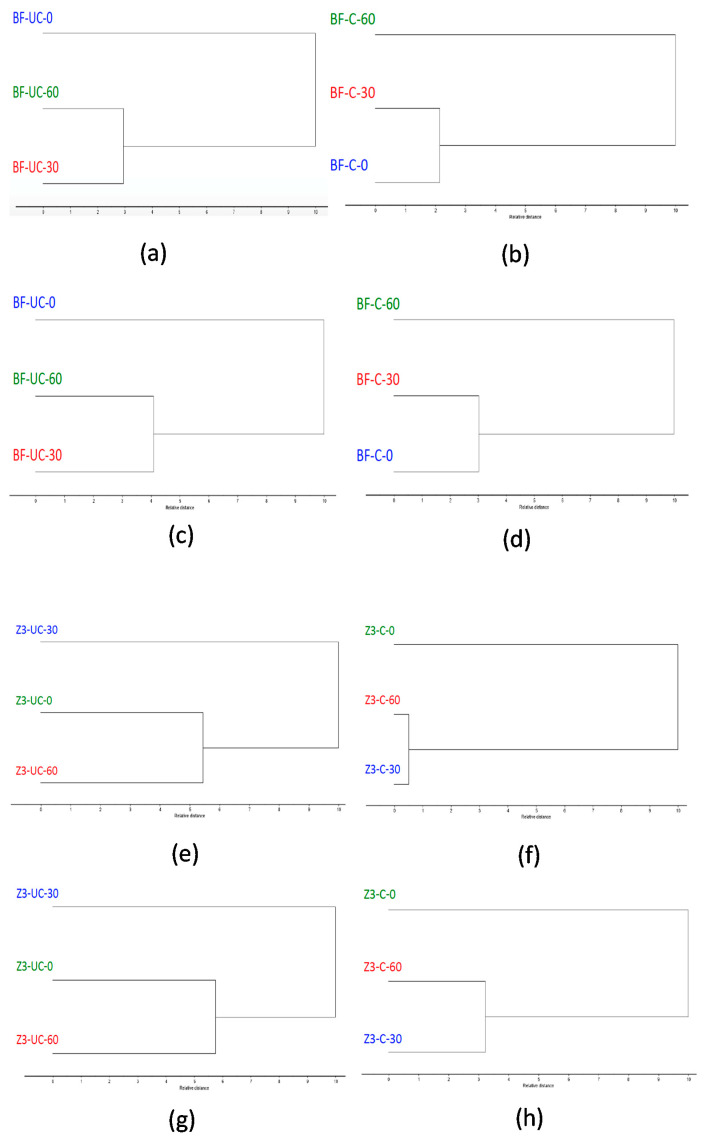
Cluster analysis of Filtek Bulk-fill composite showing uncured and cured at spectral range 1590–1650 cm^−1^ (**a**,**b**), uncured and cured at spectral range 1700–1750 cm^−1^ (**c**,**d**). Cluster analysis of Filtek Z350 composite showing uncured and cured at spectral range 1590–1650 cm^−1^ (**e**,**f**), uncured and cured at spectral range 1700–1750 cm^−1^ (**g**,**h**). At both the spectral ranges, there was a distinct separation without ultrasonic treatment from 30 s and 60 s while in the uncured state. However once cured, Filtek Bulk-fill showed that the control branched closer to the 30 s treated composite, whereby Filtek Z350 showed that the control was closer to both the 30 s and 60 s ultrasonic treatments.

Cluster analyses of Z250 and SDR are shown in Figure 7a–h and Z350 and Bulk-fill are presented in Figure 8a–h. At both the spectral ranges, cluster analysis of Z250 dental composites showed that without curing, control (without ultrasonic treated) was branched closer to 30 s treated composites at the 1700–1750 cm^−1^ spectral range and the 60 s treated composite at the 1590–1650 cm^−1^ spectral range (Figure 7a,c). However, there was a different pattern of separation where the non-ultrasonically treated composite was distinct from 30 s and 60 s while in the cured state (Figure 7b,d).

Cluster analyses of SDR (Figure 7e,g) and Bulk-fill (Figure 8a,c) composites demonstrated a distinct separation of composites without ultrasonic treatment from 30 s and 60 s while in the uncured state. However, once cured, non-ultrasonically treated composites were branched closer to the 30 s treated composites. Cluster analysis of Z350 composites showed a similar separation pattern with Z250. The spectra showed that without curing, the control was branched closer to 60 s (Figure 8e,g). However, the control (without ultrasonically treated composites) was distinct from 30 s and 60 s while in the cured state (Figure 8f,h).

**Figure 9:**
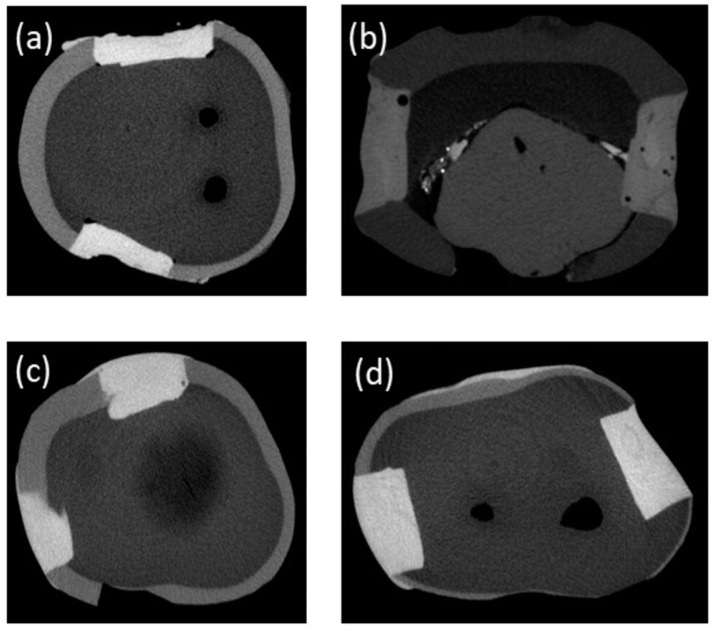
Micro-CT images showing representative samples of (**a**) Filtek Bulk-fill, (**b**) SDR, (**c**) Filtek Z250, and (**d**) Filtek Z350 after 60 s of ultrasonic activation. The images show presence of voids within the composite restorations, whereby SDR appears with maximum voids compared to the other groups.

**Figure 10:**
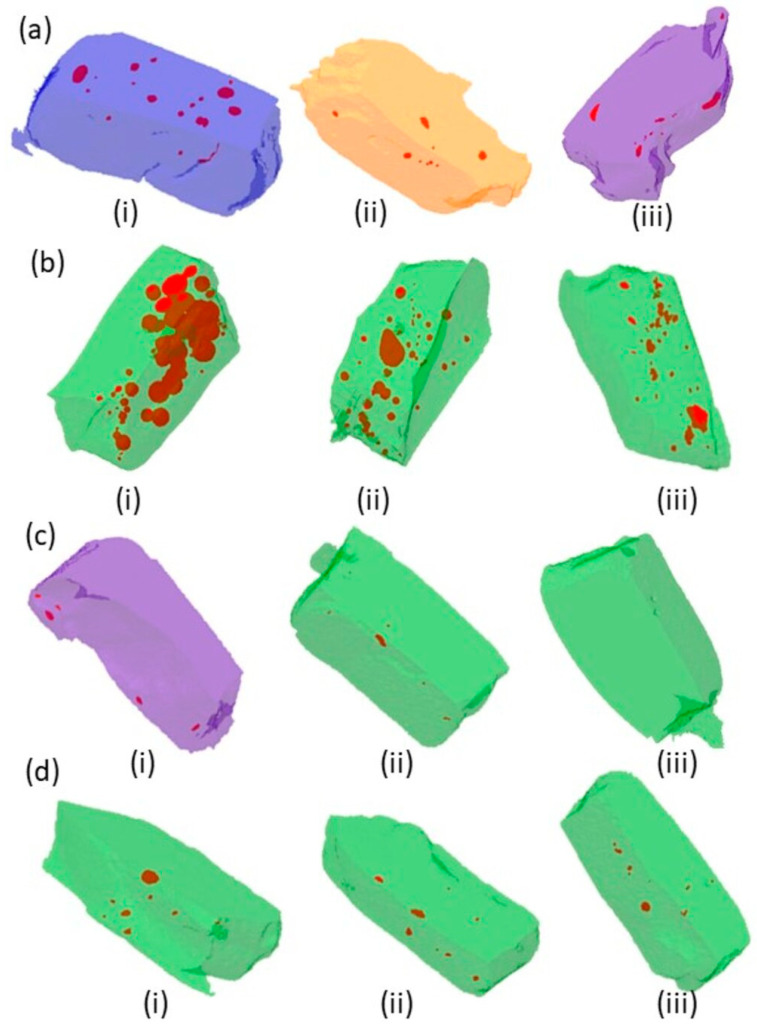
3D micro-CT analysis showing the presence of voids of (**a**) Filtek Bulk-fill, (**b**) SDR, (**c**) Filtek Z250, and (**d**) Filtek Z350; (**i**) without ultrasonic application, (**ii**) with 30 s ultrasonic application, and (**iii**) 60 s ultrasonic application.

The comparative void volume (mm^3^) with and without application of ultrasonic scaler is tabulated in Table 2 and the 3D morphological pattern and presence of voids are presented in Figure 9 and Figure 10.

Bulk-fill composites showed non-significant differences between control and 30 s groups (*p* = 0.07), whereas there was a significant difference between control and 60 s (*p* = 0.03) and a non-significant difference between 30 s and 60 s groups (*p* = 0.07). For SDR, reduction in void volume was observed, whereby a significant difference was observed between control and 30 s (*p* = 0.02) and 60 s (*p* = 0.008); however, a non-significant difference (*p* = 0.14) was found between the 30 s and 60 s groups. A non-linear behavior was observed for Z250 and Z350 where with Z350, the void volumes reduced with 30 s ultrasonic application; however, the voids were increased with 60 s sonic vibrations. The difference between control and 60 s was non-significant (*p* = 0.86). A similar trend was observed in the Z250 group, whereby there was a slight increase in voids with 30 s (*p* = 0.33) compared to control and 60 s; however, the difference was non-significant (*p* = 0.64).

## 4. Discussion

Dental composite materials have been revolutionized with continuous advancements and improvisations. In addition to micro- and nano-hybrid resin composites, flowable and bulk-fill composites have gained interest among dentists due to greater light penetration that allows curing of 4 to 5 mm and potentially saves clinical placement time [46,47]. All these composites differ from each other in terms of their chemical composition or the filler type and filler amount [48]. The concerns associated with all these types of composites are polymerization shrinkage and the presence of uncured monomer. It is reported that after photo-curing the polymerization reaction is not complete and methacrylic compounds are released into the oral cavity tissues where they may cause local adverse effects [13].

In this study, an attempt was taken to use an ultrasonic scaler as preheating tool with the concept that ultrasonic application can increase the polymerization reaction subsequently degree of conversion. Moreover, it can help to reduce the presence of voids in composites.

The vibration mode and amplitude of ultrasonic instruments depend on the frequency and power supply of the devices [30]. In this study, the frequency, power, and time of ultrasonic application were optimized on the basis of a pilot study. The frequency and time of less than 15 Hz and 30 s, respectively, did not perform differently for composites. The composites were placed in a prepared tooth cavity to evaluate the degree of conversion and void volume to mimic the clinical situation. The mold (tooth cavity) selection conducted in this study might be explained by the fact that the ultrasonic waves represent a mechanical propagation of energy through a medium [30]. A previous study analyzed different molds i.e., Teflon, enamel, dentin, pulp, and the reported ultrasonic wave propagations were 1518, 3100, 1900, and 1570 m/s respectively [49]. Based on this reported data, the human teeth mold should be used to simulate the clinical scenario.

The viscosity of the monomer, as well as the flexibility in its chemical structure, are the principal features which can influence the degree of conversion. Similar findings were observed in this study, where SDR with low viscosity showed higher DC% initially compared to other groups; however, after ultrasonic application, all groups except SDR showed an increase in DC%. SDR showed a decrease in the DC after the application of ultrasonic vibrations. The change became more evident when the application of the ultrasonic vibrations increased from 30 s to 60 s which decreased its DC% significantly. Although with the control group the SDR showed more DC than the rest of the three materials. The study showed a maximum increase of temperature with SDR, however, burning of SDR was observed with 60 s ultrasonic application. The burning of resins affected the polymerization process of SDR. Among these groups, SDR has less weight percentage of fillers (barium borosilicate, 68 wt.%) [39], whereby the filler weight percentages in Filtek Bulk-fill, Filtek Z250, and Filtek Z350 are 74 wt.%, 82 wt.%, and 78.5, wt.%, respectively [37]. It has been reported that the fillers have the ability to absorb external energy and act as thermal insulators. It revealed that the application of ultrasonic vibrations has significantly affected the structural properties of the SDR. This could be due to the absence of high molecular weight resins e.g., bis-GMA and less weight percentage of fillers compared to other composites. Thus, for SDR, ultrasonic application for more than 30 s is not recommended. In this study, the temperature of Z250 and Z350 was raised to around 45–46 °C with 60 s ultrasonic application, respectively, and 52.76 °C of Bulk-fill composite. With the increase in temperature, the viscosity of the composite paste reduces due to more mobility of free radicals and propagating polymer chains resulting in a more complete polymerization reaction and greater cross-linking. However, there are certainly other factors than viscosity which influence the degree of conversions such as resin composition, filler content, and amount. This present study also showed that composites with high filler percentages with high molecular weight composites showed better responses to preheating with the ultrasonic application. High molecular weight with entangled polymeric chain length responds to preheating and allows molecules the freedom to move [50]. It is expected that ultrasound can cause acceleration within the network and may excite filler particles. The sonic excitation may de-cluster particles and reduce the porosities within the network [35]. The use of vibration energy utilizing the thixotropic effect may cause changes in the filler/matrix distribution [51]. Thus, variation in chemistry and composition of resin composites responds differently to preheating procedures.

There was a significant difference between the groups used in the study with and without using the ultrasonic application. A significant difference has also been noticed while the time of ultrasonic application was increased. The change in DC% of Z350 was more compared to Bulk-fill while the Z250 showed more conversion than the Z350, not only in the control group but also after the ultrasonic applications. While analyzing at both ends of the cluster of the materials, there was not much significant difference between the control as well as the 30 s group when comparing the Z250 composite and it showed a unique pattern of separation. The point of concern is that the control group was showing the closest spectral range when compared with the experimental group for the designated spectral range.

The cluster analysis of the Z350 group exhibited almost similar properties and outcomes when compared with the Z250 group, in terms of spectral ranges. While analyzing the clusters of Bulk-fill and SDR (control group), both exhibited a clear composite separation without ultrasonic application without curing, while, once they were cured, showed some close relation with the 30 s group. It is reported in the literature that the monomer conversion is directly related to the polymerization shrinkage and risk of interfacial gap formations [52]. However, in this present study, the micro-CT analysis showed a reduction in voids and gaps with ultrasonic applications. These results are in accordance with a previous study [53] where the lowest volumetric polymerization shrinkage was found with the highest degree of conversion. Many factors can affect the volumetric polymerization shrinkage of resin-based composite such as the filler content, resin matrix composition, molecular weight of the resins, flow and rate of the modulus of the resin, and polymerization methods [54,55].

In this study, change in void volume was also assessed with 3D micro-CT. The micro-CT is a non-destructive analytical tool, which can provide accurate visualization for marginal adaptation and volumetric changes [56]. The presence of voids in restorations might be due to condensing technique and incremental placement of restorations. The high viscosity of the composite restoration hinders the proper condensation [57]. Previously, it has been reported that the voids have been observed in bulk-fill and flowable composites ampules. The presence of voids in restoration can cause problems in the physical and mechanical properties of the restoration [58]. In the present study, an ultrasonic scaler was applied to evaluate the change in the void volume. It is expected that an increase in temperature reduced the presence of voids. All composite groups showed a reduction in the void volume, which might be due to a reduction in the viscosity of the composites. In the control group, SDR and Bulk-fill composites showed more voids compared to the other groups, which might be pre-existing as a result of manufacturing methods as reported previously or might be related to technical difficulties in placing flowable and bulk-fill composites. These findings are in accordance with previous studies where porosities were observed after flowable composite placement [59,60]. The smaller filler percentage and high amount of resin makes them more difficult to pack into cavity preparations and increases the technical difficulty of removing microbubbles during the manufacturing process. To overcome this issue, an ultrasonic scaler was applied, which on one side reduces the viscosity but may also reduce microbubbles due to vibrational motion within the resin system. Both SDR and Bulk-fill showed a significant reduction in the void volume with 60 s ultrasonic application compared to the other composites. Minimum voids were observed in Z350 and significant reduction was observed with 30 s application, however, the voids’ volume increased with the increase in ultrasonic application time. Similarly, Z250 showed non-linear behavior, whereby with 30 s application the increase in void volume was observed, however, it was reduced with 60 s application. All composite groups respond differently to ultrasonic application due to difference in their compositions. The findings of this study showed that variation in composites’ composition does not impart predictive information regarding the reduction in void volumes. However, it is expected that not only resin combination and filler amount, but also the size of fillers may affect the properties of the material. Z350 is a nano-hybrid and Z250 is a micro-hybrid composite.

In this study, an ultrasonic scaler was used to produce heat with the concept that preheating procedure can be done at the chair side. Previously, composite restorations have been preheated in a composite warming tray, a water bath, or a composite warmer [61,62]. These procedures are technique sensitive and time consuming. Moreover, the effect of preheating is still questionable due to the rate of ambient cooling. It is reported that the temperature dropped 50% within 2 min of removing a composite from a heating device [63]. Therefore, preheating away from the chair side during clinical procedures cannot provide the required results. In this study, preheating of the composite was performed within the tooth cavity and an increase in temperature was observed, which can increase the temperature within the cavity. However, it is reported that the use of preheated composites (set to 54 °C or 60 °C) did not produce high in vitro intrapulpal temperatures, whereby the rise in temperature inside the pulp was 0.8 °C while the rise due to light-curing was 5 °C [64]. Thus, the rise in temperature due to ultrasonic application was in an acceptable range. In this study, the mechanical properties and shrinkage behavior were not evaluated after ultrasonic application. In the literature, studies report contradictory data; one study [65] mentioned that preheating of composites to relatively high temperatures of 54 °C and 88 °C increased the volumetric shrinkage, whereas another study [66] opposed this and reported that preheating (68 °C) of the composite did not negatively affect the shrinkage. The rise in temperature with ultrasonic application varied with different types of composites and with 30 s and 60 s ultrasonic application, the maximum reported temperatures were 46 °C and 62 °C for SDR. However, as per the results of this current study, it is not recommended to use a 60 s ultrasonic application for SDR. Nada et al. 2011 [67] also reported that the effect of preheating on composites’ mechanical properties is material dependent.

The types of fillers can also influence the physical and mechanical properties of restorative materials. In this study, the used composites mainly contained barium borosilicate, zirconia, and nanoclusters of silica. Recently, bioceramic-based materials, fluoride, and remineralizing agents have been investigated in experimental dental restorative materials. These materials have a significant effect on the physical and mechanical properties of the restorative materials and can alter the restorative/tooth interface [68,69]. Clinically, filler contents, types of fillers, and morphology of fillers can also influence the results. Therefore, future in vitro and clinical studies are needed to evaluate these unexplored variables. Another limitation of this study is that other types of flowable, bulk, nano-hybrid and micro-hybrid commercial composites should be evaluated to get better comparative results. Previously, the marginal adaptation and water sorption properties were evaluated after applying the preheated composites [70,71]. Therefore, it is recommended that the interfacial linkage, mechanical properties (such as flexural strength and compressive strength), and water sorption characteristics should be evaluated in the future.

## 5. Conclusions

Within the limitations of this study, it is concluded that the effect of ultrasonic scaler was observed in the degree of conversion, and change in the void volume of composites was also observed. An increase in temperature was observed with the ultrasonic application. SDR showed the highest increase in temperature followed by Bulk-fill, Z350, and Z250. Bulk-fill, Z350, and Z250 have shown an increase in the degree of conversion with an increase in time of ultrasonic application; however, SDR showed non-linear behavior. The cluster analysis showed excellent separation invariances. The micro-CT analysis showed a reduction in void volume for SDR and Bulk-fill with 30 s and 60 s ultrasonic application, whereby Z250 and Z350 showed non-linear behavior. It is suggested that 60 s application of ultrasonic scaler can provide better results in terms of DC and reduce the void volume of composites. The frequency and time of the ultrasonication is an important factor to consider because it can affect the properties of the material.

## Figures and Tables

**Table 1:** Composition of commercially used dental restorative materials.

Commercial Name	Type	Composition	References
Filtek^TM^ Z250 (Lot No: NB29540; 3M ESPE, St. Paul, MN, USA)	Micro-hybrid	bis-GMA, UDMA, and bis-EMA resins Zirconia/Silica (82 wt.%)	[37]
Filtek^TM^ Bulk-Fill (Lot No: N7060903M ESPE, St. Paul, MN, USA)	Bulk-fill nanocomposite	ERGP-DMA, diurethane-DMA, and 1,12-dodecane-DMA zirconia/silica cluster filler, ytterbium trifluoride (74 wt.%)	[38]
Filtek^TM^ Z350 (Lot No: N582322; 3M ESPE, St. Paul, MN, USA)	Nano-hybrid	bis-GMA, UDMA, TEGDMA and bis-EMA resins, PEGDMA Nanoclusters of silica (78.5 wt.%)	[37]
SureFil^®^SDR^TM^ flow (Lot No: 1705000885: DENTSPLY, Konstanz, Germany)	Flowable	UDMA, TEGDMA, Di-methacrylate resin, trimethacrylate resins barium-alumino-fluoro-borosilicate glass; silanated strontium alumino-fluoro-silicate glass; surface treated fume silicas; ytterbium fluoride; synthetic inorganic iron oxide pigments and titanium dioxide (68 wt.%)	[39]

bis-GMA = bisphenol A-glycidyl methacrylate; UDMA = urethane dimethacrylate; TEGDMA = triethylene glycol dimethacrylate; Bis-EMA = bisphenol ethyl methacrylate; PEGDMA = polyethyl glycol dimethacrylate; DMA = dimethacrylate.

**Table 2:** Mean (SD) void volume (mm^3^) of composite groups without (CT) and with ultrasonic application of 30 s (US-30) and 60 s (US-60).

Composites	CT	US-30	US-60
Bulk-fill	0.479 ± 0.114	0.180 ± 0.036	0.077 ± 0.021
SDR	0.821 ± 0.096	0.214 ± 0.089	0.053 ± 0.038
Z250	0.038 ± 0.007	0.047 ± 0.006	0.035 ± 0.001
Z350	0.015 ± 0.007	0.006 ± 0.005	0.014 ± 0.005

## Data Availability

Not applicable.

## References

[1] Al-Nahedh H.N., El-Hejazi A.A., Habib S.R. (2020). Knowledge and Attitude of Dentists and Patients Toward Use and Health Safety of Dental Amalgam in Saudi Arabia. Eur. J. Dent..

[2] Mackey T.K., Contreras J.T., Liang B.A. (2014). The Minamata Convention on Mercury: Attempting to address the global controversy of dental amalgam use and mercury waste disposal. Sci. Total Environ..

[3] Quek Wei QIAN C., Amir Wan Ahmad W.M., Yusop N., Ghazalli N.F., Samsudin N.A. (2020). Anterior Composite Resin Restoration Preference Among General Dental Practitioners in Penang Island, Malaysia. Int. J. Med. Dent..

[4] Khan A.S., Ur Rehman S., AlMaimouni Y.K., Ahmad S., Khan M., Ashiq M. (2020). Bibliometric Analysis of Literature Published on Antibacterial Dental Adhesive from 1996–2020. Polymers.

[5] Rickman L.J., Padipatvuthikul P., Chee B. (2011). Clinical applications of preheated hybrid resin composite. Br. Dent. J..

[6] Soares C.J., Faria-E-Silva A.L., Rodrigues M.d.P., Vilela A.B.F., Pfeifer C.S., Tantbirojn D., Versluis A. (2017). Polymerization shrinkage stress of composite resins and resin cements—What do we need to know?. Braz. Oral Res..

[7] Celik C., Cehreli S.B., Arhun N. (2015). Resin composite repair: Quantitative microleakage evaluation of resin-resin and resin-tooth interfaces with different surface treatments. Eur. J. Dent..

[8] Radhika M., Sajjan G.S., Kumaraswamy B.N., Mittal N. (2010). Effect of different placement techniques on marginal microleakage of deep class-II cavities restored with two composite resin formulations. J. Conserv. Dent..

[9] Tauböck T.T., Jäger F., Attin T. (2019). Polymerization shrinkage and shrinkage force kinetics of high-and low-viscosity dimethacrylate-and ormocer-based bulk-fill resin composites. Odontology.

[10] Bayne S.C., Thompson J.Y., Swift E.J., Stamatiades P., Wilkerson M. (1998). A characterization of first-generation flowable composites. J. Am. Dent. Assoc..

[11] Kaisarly D., El Gezawi M., Keßler A., Rösch P., Kunzelmann K.-H. (2021). Shrinkage vectors in flowable bulk-fill and conventional composites: Bulk versus incremental application. Clin. Oral Investig..

[12] Rizzante F.A.P., Mondelli R.F.L., Furuse A.Y., Borges A.F.S., Mendonça G., Ishikiriama S.K. (2019). Shrinkage stress and elastic modulus assessment of bulk-fill composites. J. Appl. Oral Sci..

[13] Khalid H., Syed M.R., Rahbar M.I., Iqbal H., Ahmad S., Kaleem M., Matinlinna J.P., Khan A.S. (2018). Effect of nano-bioceramics on monomer leaching and degree of conversion of resin-based composites. Dent. Mater. J..

[14] Fronza B.M., Rueggeberg F.A., Braga R.R., Mogilevych B., Soares L.E.S., Martin A.A., Ambrosano G., Giannini M. (2015). Monomer conversion, microhardness, internal marginal adaptation, and shrinkage stress of bulk-fill resin composites. Dent. Mater..

[15] Bakhsh T.A., Sadr A., Shimada Y., Turkistani A., Abuljadayel R., Tagami J. (2020). Does lining class-II cavities with flowable composite improve the interfacial adaptation?. J. Adhes. Sci. Technol..

[16] Sarapultseva M., Sarapultsev A. (2019). Flowable Bulk-Fill Materials Compared to Nano Ceramic Composites for Class I Cavities Restorations in Primary Molars: A Two-Year Prospective Case-Control Study. Dent. J..

[17] Nocca G., De Palma F., Minucci A., De Sole P., Martorana G., Callà C., Morlacchi C., Gozzo M., Gambarini G., Chimenti C. (2007). Alterations of energy metabolism and glutathione levels of HL-60 cells induced by methacrylates present in composite resins. J. Dent..

[18] Reichl F.-X., Esters M., Simon S., Seiss M., Kehe K., Kleinsasser N., Folwaczny M., Glas J., Hickel R. (2006). Cell death effects of resin-based dental material compounds and mercurials in human gingival fibroblasts. Arch. Toxicol..

[19] Michelsen V.B., Kopperud H.B., Lygre G.B., Björkman L., Jensen E., Kleven I.S., Svahn J., Lygre H. (2012). Detection and quantification of monomers in unstimulated whole saliva after treatment with resin-based composite fillings in vivo. Eur. J. Oral Sci..

[20] Moldovan M., Balazsi R., Soanca A., Roman A., Sarosi C., Prodan D., Vlassa M., Cojocaru I., Saceleanu V., Cristescu I. (2019). Evaluation of the Degree of Conversion, Residual Monomers and Mechanical Properties of Some Light-Cured Dental Resin Composites. Materials.

[21] Halvorson R.H., Erickson R.L., Davidson C.L. (2003). The effect of filler and silane content on conversion of resin-based composite. Dent. Mater..

[22] Tuna E., Aktoren O., Oshida Y., Gencay K. (2010). Elution of residual monomers from dental composite materials. Eur. J. Paediatr Dent..

[23] Marcondes R.L., Lima V.P., Barbon F.J., Isolan C.P., Carvalho M.A., Salvador M.V., Lima A.F., Moraes R.R. (2020). Viscosity and thermal kinetics of 10 preheated restorative resin composites and effect of ultrasound energy on film thickness. Dent. Mater. Off. Publ. Acad. Dent. Mater..

[24] Ahn K.H., Lim S., Kum K.Y., Chang S.W. (2015). Effect of preheating on the viscoelastic properties of dental composite under different deformation conditions. Dent. Mater. J..

[25] Prasanna N., Pallavi Reddy Y., Kavitha S., Lakshmi Narayanan L. (2007). Degree of conversion and residual stress of preheated and room-temperature composites. Indian J. Dent. Res. Off. Publ. Indian Soc. Dent. Res..

[26] Elkaffass A.-A., Eltoukhy R.-I., Elnegoly S.-A.-E., Mahmoud S.-H. (2020). Influence of preheating on mechanical and surface properties of nanofilled resin composites. J. Clin. Exp. Dent..

[27] Daronch M., Rueggeberg F.A., De Goes M.F. (2005). Monomer Conversion of Pre-heated Composite. J. Dent. Res..

[28] Daronch M., Rueggeberg F.A., De Goes M.F., Giudici R. (2006). Polymerization kinetics of pre-heated composite. J. Dent. Res..

[29] Yao Y., Pan Y., Liu S. (2020). Power ultrasound and its applications: A state-of-the-art review. Ultrason. Sonochemistry.

[30] Chen Y.-L., Chang H.-H., Chiang Y.-C., Lin C.-P. (2013). Application and development of ultrasonics in dentistry. J. Formos. Med. Assoc..

[31] Cantoro A., Goracci C., Coniglio I., Magni E., Polimeni A., Ferrari M. (2011). Influence of ultrasound application on inlays luting with self-adhesive resin cements. Clin. Oral Investig..

[32] Kawashima H., Sato S., Kishida M., Ito K. (2007). A comparison of root surface instrumentation using two piezoelectric ultrasonic scalers and a hand scaler in vivo. J. Periodontal Res..

[33] Aydin C., Inan U., Gultekin M. (2012). Comparison of the shaping ability of Twisted Files with ProTaper and RevoS nickel-titanium instruments in simulated canals. J. Dental Sci..

[34] Coldebella C.R., Santos-Pinto L., Zuanon A.C.C. (2011). Effect of ultrasonic excitation on the porosity of glass ionomer cement: A scanning electron microscope evaluation. Microsc. Res. Tech..

[35] Barata T.J.E., Bresciani E., Adachi A., Fagundes T.C., Carvalho C.A.R., Navarro M.F.L. (2008). Influence of ultrasonic setting on compressive and diametral tensile strengths of glass ionomer cements. Mater. Res..

[36] Vanderlei A.D., Borges A.L.S., Cavalcanti B.N., Rode S.M. (2008). Ultrasonic versus high-speed cavity preparation: Analysis of increases in pulpal temperature and time to complete preparation. J. Prosthet. Dent..

[37] Monteiro G.Q.d.M., Montes M.A.J.R. (2010). Evaluation of linear polymerization shrinkage, flexural strength and modulus of elasticity of dental composites. Mater. Res..

[38] Camassari J.R., Correr-Sobrinho L., Correr A.B., Puppin-Rontani J., Stipp R.N., Puppin-Rontani R.M., Paula A.B. (2020). Physical-Mechanical Properties of Bulk Fill Composites Submitted to Biodegradation by Streptococcus mutans. Braz. Dent. J..

[39] Lassila L., Säilynoja E., Prinssi R., Vallittu P., Garoushi S. (2019). Characterization of a new fiber-reinforced flowable composite. Odontology.

[40] Secilmis A., Dilber E., Gokmen F., Ozturk N., Telatar T. (2011). Effects of storage solutions on mineral contents of dentin. J. Dent. Sci..

[41] Firzok H., Zahid S., Asad S., Manzoor F., Khan A.S., Shah A.T. (2019). Sol-gel derived fluoridated and non-fluoridated bioactive glass ceramics-based dental adhesives: Compositional effect on re-mineralization around orthodontic brackets. J. Non Cryst. Solids.

[42] Luong E., Shayegan A. (2018). Assessment of microleakage of class V restored by resin composite and resin-modified glass ionomer and pit and fissure resin-based sealants following Er:YAG laser conditioning and acid etching: In vitro study. Clin. Cosmet Investig Dent..

[43] Lemeshow S., Lwanga S. (1991). Sample Size Determination in Health Studies.

[44] Sakaguchi R., Ferracane J., Powers J. (2019). Chapter 9—Restorative Materials: Resin Composites and Polymers. Craig’s Restorative Dental Materials.

[45] Nayyer M., Zahid S., Hassan S.H., Mian S.A., Mehmood S., Khan H.A., Kaleem M., Zafar M.S., Khan A.S. (2018). Comparative abrasive wear resistance and surface analysis of dental resin-based materials. Eur. J. Dent..

[46] Bayne S.C., Ferracane J.L., Marshall G.W., Marshall S.J., van Noort R. (2019). The Evolution of Dental Materials over the Past Century: Silver and Gold to Tooth Color and Beyond. J. Dent. Res..

[47] Arbildo-Vega H.I., Lapinska B., Panda S., Lamas-Lara C., Khan A.S., Lukomska-Szymanska M. (2020). Clinical Effectiveness of Bulk-Fill and Conventional Resin Composite Restorations: Systematic Review and Meta-Analysis. Polymers.

[48] Ilie N., Hickel R. (2011). Resin composite restorative materials. Aust. Dent. J..

[49] Ghorayeb S.R., Xue T., Lord W. (1998). A finite element study of ultrasonic wave propagation in a tooth phantom. J. Dent. Res..

[50] Dionysopoulos D., Tolidis K., Gerasimou P., Koliniotou-Koumpia E. (2014). Effect of preheating on the film thickness of contemporary composite restorative materials. J. Dent. Sci..

[51] Schmidlin P.R., Zehnder M., Schlup-Mityko C., Göhring T.N. (2005). Interface evaluation after manual and ultrasonic insertion of standardized class I inlays using composite resin materials of different viscosity. Acta Odontol. Scand..

[52] Kaisarly D., Gezawi M.E. (2016). Polymerization shrinkage assessment of dental resin composites: A literature review. Odontology.

[53] Salem H.N., Hefnawy S.M., Nagi S.M. (2019). Degree of Conversion and Polymerization Shrinkage of Low Shrinkage Bulk-Fill Resin Composites. Contemp Clin. Dent..

[54] Garoushi S., Vallittu P.K., Watts D.C., Lassila L.V.J. (2008). Polymerization shrinkage of experimental short glass fiber-reinforced composite with semi-inter penetrating polymer network matrix. Dent. Mater..

[55] Almeida Junior L.J.d.S., Penha K.J.d.S., Souza A.F., Lula E.C.O., Magalhães F.C., Lima D.M., Firoozmand L.M. (2017). Is there correlation between polymerization shrinkage, gap formation, and void in bulk fill composites? A μCT study. Braz. Oral Res..

[56] Kakaboura A., Rahiotis C., Watts D., Silikas N., Eliades G. (2007). 3D-marginal adaptation versus setting shrinkage in light-cured microhybrid resin composites. Dent. Mater. Off. Publ. Acad. Dent. Mater..

[57] Opdam N.J., Roeters J.J., Joosten M., Veeke O. (2002). Porosities and voids in Class I restorations placed by six operators using a packable or syringable composite. Dent. Mater. Off. Publ. Acad. Dent. Mater..

[58] Mulder R., Mohammed N., Du Plessis A., Le Roux S. (2017). A pilot study investigating the presence of voids in bulk fill flowable composites. South. Afr. Dent. J..

[59] Chuang S.-F., Liu J.-K., Chao C.-C., Liao F.-P., Chen Y.-H.M. (2001). Effects of flowable composite lining and operator experience on microleakage and internal voids in class II composite restorations. J. Prosthet. Dent..

[60] Opdam N., Roeters J., de Boer T., Pesschier D., Bronkhorst E. (2003). Voids and porosities in class I micropreparations filled with various resin composites. Oper. Dent..

[61] Mohammadi N., Jafari-Navimipour E., Kimyai S., Ajami A.-A., Bahari M., Ansarin M., Ansarin M. (2016). Effect of pre-heating on the mechanical properties of silorane-based and methacrylate-based composites. J. Clin. Exp. Dent..

[62] Jin M.U. (2013). Prepare the pre-heated composite resin. Restor. Dent. Endod..

[63] Daronch M., Rueggeberg F.A., Moss L., de Goes M.F. (2006). Clinically relevant issues related to preheating composites. J. Esthet. Restor. Dent. Off. Publ. Am. Acad. Esthet. Dent..

[64] Daronch M., Rueggeberg F.A., Hall G., De Goes M.F. (2007). Effect of composite temperature on in vitro intrapulpal temperature rise. Dent. Mater. Off. Publ. Acad. Dent. Mater..

[65] Walter R., Swift E.J., Sheikh H., Ferracane J.L. (2009). Effects of temperature on composite resin shrinkage. Quintessence Int..

[66] Tantbirojn D., Chongvisal S., Augustson D.G., Versluis A. (2011). Hardness and postgel shrinkage of preheated composites. Quintessence Int..

[67] Nada K., El-Mowafy O. (2011). Effect of precuring warming on mechanical properties of restorative composites. Int. J. Dent..

[68] Bijle M.N., Ekambaram M., Lo E., Yiu C. (2020). Physicochemical Characteristics of Arginine Enriched NaF Varnish: An In Vitro Study. Polymers.

[69] Scribante A., Dermenaki Farahani M.R., Marino G., Matera C., Rodriguez Y.B.R., Lanteri V., Butera A. (2020). Biomimetic Effect of Nano-Hydroxyapatite in Demineralized Enamel before Orthodontic Bonding of Brackets and Attachments: Visual, Adhesion Strength, and Hardness in In Vitro Tests. Biomed. Res. Int..

[70] Darabi F., Tayefeh-Davalloo R., Tavangar S.-M., Naser-Alavi F., Boorboo-Shirazi M. (2020). The effect of composite resin preheating on marginal adaptation of class II restorations. J. Clin. Exp. Dent..

[71] Kaboorani A., Englund K.R. (2011). Water sorption and mechanical performance of preheated wood/thermoplastic composites. J. Compos. Mater..

